# Mid-Term Results following Traumatic Knee Joint Dislocation

**DOI:** 10.3390/jcm12010266

**Published:** 2022-12-29

**Authors:** Julius Watrinet, Christian von Rüden, Stephan Regenbogen, Andreas Brand, Markus Bormann, Fabian M. Stuby, Julian Fürmetz

**Affiliations:** 1 Department Trauma Surgery, BG Unfallklinik Murnau, 82418 Murnau, Germany; 2 Institute for Biomechanics, Paracelsus Medical University, 5020 Salzburg, Austria; 3 Institute of Biomechanics, BG Trauma Center Murnau, 82418 Murnau, Germany; 4 Department of Orthopaedics and Trauma Surgery, Musculoskeletal University Center Munich (MUM), University Hospital, LMU Munich, 81377 Munich, Germany

**Keywords:** knee, knee dislocation, ACL, PCL, external fixation, arthrofibrosis

## Abstract

Purpose: Although treatment strategies of knee joint dislocations have evolved, there is still no consensus on the best method and timing. New therapeutic concepts suggest that early one-stage treatment, including suturing and bracing of the cruciate ligaments in acute knee joint dislocation, are leading to improved functional results. This study aimed to evaluate the midterm functional outcome following traumatic knee joint dislocation and to determine whether the outcome is influenced by the surgical management, patient habitus or concomitant injuries. Methods: In this retrospective single center study, 38 patients with acute Schenck type II to IV knee dislocations were treated over an eight-year period in a level I trauma center. At follow-up, various clinical scores, such as the International Knee Documentation Committee (IKDC) Score, Lysholm Score, and Tegner Activity Scale (TAS), and individual questions about rehabilitation and activity levels of 38 patients were evaluated. Results: Mean follow-up was 5.5 ± 2.7 years. The mean IKDC Score was 65.6 ± 15.7 points, the average Lysholm Score was 70.5 ± 16.4 points and the median TAS was 4 (0–7), resulting in a loss of activity of 2 (range 0–6) points. There was no significant difference between a one-stage treatment compared to a two-stage approach. Ligament reconstruction of the ACL in a two-stage approach was required in only 33.3%. Further operations (early and late) were performed in 37% of cases. Being overweight was associated with more complications and worse outcomes, and external fixation with arthrofibrosis. Conclusions: Knee dislocation is a severe trauma that often leads to a prolonged loss of function and increased knee pain over years, affecting the patient’s activity. Clinical outcome is influenced significantly by concomitant injuries. Severe cases with initial external fixation are associated with a higher risk of knee stiffness and should be considered during rehabilitation. Obese patients present a challenge due to higher complication rates and lower postoperative knee function. Level of evidence: Retrospective single center study, level III.

## 1. Introduction

Acute knee dislocation is a severe injury requiring immediate und multidisciplinary diagnosis and treatment. The incidence is about 0.001 to 0.02%, but the actual number remains unknown, as some cases go undetected [1,2,3].

High velocity (HV) trauma is present in 50% of cases and patients are often polytraumatized, while 40% of injuries occur at low velocity (LV) [2,4]. The ultra-low-velocity (ULV) trauma is classified as an additional entity, which occurs predominantly in very obese patients, who are more likely to suffer from a higher incidence and severe complications [5,6,7,8]. 

Being a very complex injury, there is no evidence-based standard treatment in terms of timing, surgical approach, and techniques. There are multiple strategies with low evidence, as studies often have small sample sizes and short follow-up periods [9,10,11,12,13,14,15,16]. Conservative treatment is leading to unsatisfying results [11,17,18,19]. A two-stage strategy, where initially only peripheral structures are addressed and the reconstruction of the cruciate ligaments is delayed, is common [20,21,22,23,24,25]. The use of an external knee spanning fixator is frequent in trauma surgery and suggested for patients with arterial injury, severe instability or complex soft tissue trauma, while there is limited data on the possible disadvantages [26,27].

Some authors suggest primary suture repair, which achieves similar results to two-stage surgery [11,23,28,29]. Recent studies demonstrated good clinical results following a one-stage approach, suggesting early refixation, including the bracing of the posterior cruciate ligament (PCL), although long-term results were missing [30,31]. Bracing is shown to be superior to refixation alone, as it can withstand higher forces and leads to less elongations, demonstrated in a biomechanical study [32]. 

There is no consensus on the required treatment concept in the rehabilitation phase either. Different types of braces and rehabilitation regimes are common, while there is no evidence favoring one special protocol [9,11,12,13,23,30]. 

Complications, such as lesions to the common peroneal nerve (4–40%) (CPN), as well as to the popliteal artery (18%), are frequently seen and can change the outcome dramatically [33,34,35]. Recovery from CPN lesions with sufficient motoric function is seen in around 50% [35]. of cases. Compartment syndromes occur in small numbers, around 3%, especially in severely injured patients [36]. 

To investigate midterm functional outcome following acute knee dislocation an eight-year period at a level one trauma center was reviewed.

It was hypothesized that a one-stage approach involving early and complete restoring of knee stability, including PCL augmentation, would lead to better functional outcome and early recovery, compared to a two-stage treatment. It was also hypothesized that patients would not benefit from treatment with an external fixator.

## 2. Materials and Methods

Between June 2011 and June 2019, 120 patients with knee dislocation were treated at a level one trauma center (Table 1). Patients with Schenck type I and V knee dislocations, associated with a tibial or femoral fracture and severe trauma with limited options for trauma care, were excluded (*n* = 75). A total of 45 patients with clinical or radiological evidence of acute knee dislocation Schenck types II–IV were included. All knee dislocations were classified using the Schenck classification [30,31,35]. The minimum follow-up was 2 years. Patients with peroneal nerve or popliteal artery injuries and chain injuries of the same limb or pelvic fractures were included, unless treatment of the knee joint dislocation was limited. 

### 2.1. Surgical Management

**Figure 1:**
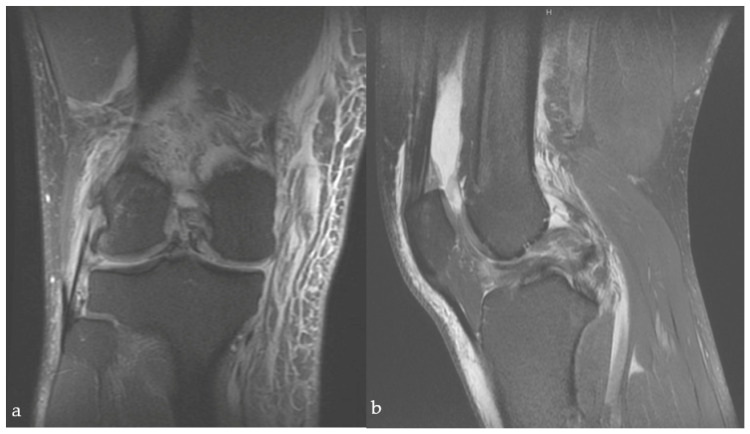
Sagittal (**a**) and frontal (**b**) view of a T2-weighted MRI scan of a right knee 2 days after KD type IV with disruption of both cruciate and bilateral bucket handle tear.

**Figure 2:**
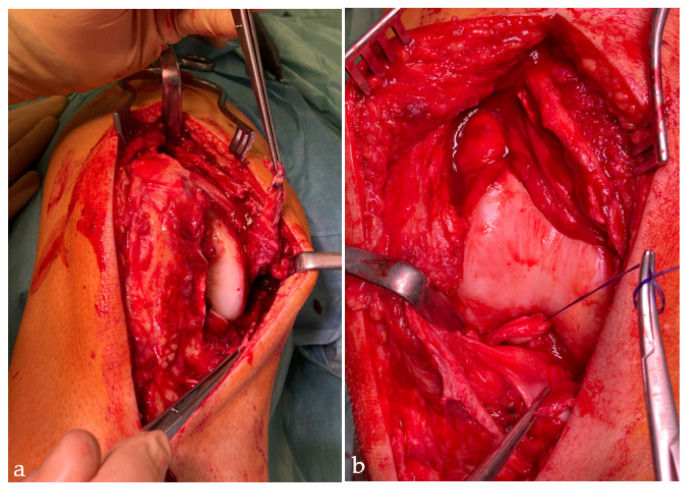
(**a**) Intraoperative view with a torn medial collateral ligament (for-ceps) and (**b**) bucket handle tear of both menisci.

**Figure 3:**
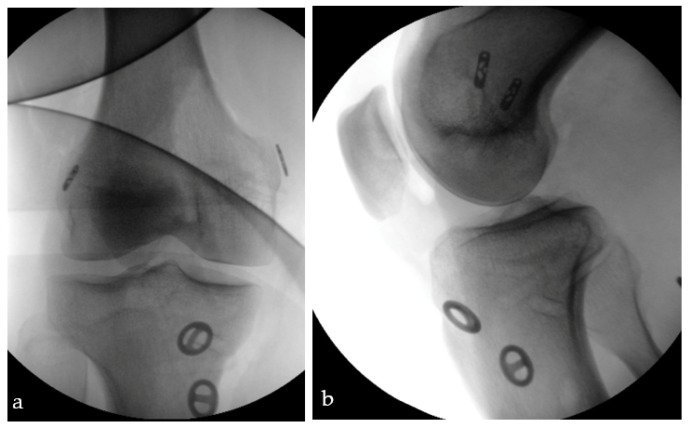
Intraoperative fluoroscopic imaging (**a**) ap-view and (**b**) lateral-view after augmentation of the ACL and PCL with cortical fixed suture tapes.

Surgical management was performed according to radiological findings in X-ray, computer-tomography and magnetic resonance imaging (Figure 1), as well as intraoperative clinical evaluation. Operative concepts were individualized according to the injury type and torn ligaments were treated when possible (Figure 2 and Figure 3). All procedures were performed by the leading consultant for knee surgery. 

### 2.2. Rehabilitation

During rehabilitation, patients generally had a stabilizing brace and had partial weight bearing, with a maximum of 20 kg, for 4 to 6 weeks. Different braces were used, most commonly a PCL brace. The range of motion was limited for 8 to 12 weeks. The rehabilitation program was highly individualized, and, in some cases, inpatient rehabilitation was recommended.

### 2.3. Follow-Up Evaluation

Patients were contacted 2 to 8 years after injury. Objective and subjective clinical outcomes were determined using the IKDC Score and the Lysholm Score to assess subjective knee function. The TAS was used to measure sports level before the injury and at follow-up [37,38,39]. Additionally, non-standardized single questions were asked. 

### 2.4. Statistical Analysis

Descriptive data (IKDC, Lysholm) were presented with mean values ± standard deviations (SDs) for continuous variables. The TAS was calculated using median and range values, and calculated by means of SPSS 26.0.0.1 program (SPSS Inc., Chicago, IL, USA). When comparing characteristics of the 2 groups, the Mann–Whitney-U test was used. A *p*-value of 0.05 or lower was considered statistically significant.

## 3. Results

### 3.1. Follow-Up and Demographics

A total of 45 patients with a mean follow-up of 5.5 ± 2.7 years were included in the study. Of these, 7 patients were lost to follow-up (15.6%). The male-to-female ratio was 2.5:1 and the mean age of patients was 49.5 ± 12.7 years (22–75).

### 3.2. Trauma Mechanism and Surgical Treatment

Twenty-eight patients suffered a mono trauma, 7 patients presented with multiple trauma and 3 patients were polytraumatized. HV trauma occurred in 20 cases, LV trauma was documented in 16 cases and 2 patients with ULV trauma were observed. 

Twenty patients received a reconstruction of all torn ligaments in one operation, while 15 patients had a two-stage procedure. Three patients only received external fixation of the knee and no further reconstruction. 

Ninety two percent of the patients were treated within 14 days after trauma, while 3 patients had delayed treatment after 29 to 142 days. If a PCL lesion was present, suturing, as well as augmentation, was performed in 60.5% (*n* = 23), while suturing alone was performed in 18.4% (*n* = 7) and conservative treatment was documented in 21.1% (*n* = 8). 

### 3.3. Functional Outcome

Overall postoperative patient reported outcome scores (IKDC and Lysholm) are displayed in Table 2. In addition, the TAS was 7 (range 3–9) points before trauma and 4 (range 0–7) points after trauma, and patients had lower activity levels that decreased over 2 (range 0–6) points. 

Clinical outcome regarding whether one- or two-staged therapy was performed, or only external fixation with no reconstruction, due to concomitant trauma, is provided below (Table 3). No significant difference was observed between patients with one-stage or two-stage procedures. 

### 3.4. Comorbidities, Vascular and Nerve Injury

Concomitant injuries, such as severe additional trauma, vascular or nerve injury, were observed in 18 cases. Clinical outcome was inferior when concomitant injuries were present, as significant differences regarding the Lysholm score (*p* < 0.05) were observed, as shown in Table 4.

Ten patients had an initial peroneal nerve injury. Of these, 5 patients suffered from a motor peroneal lesion after knee dislocation at follow-up, while 5 patients recovered. Patients with persistent CPN had worse outcomes (IKDC 53.3 ± 21.2; Lysholm score 63.2 ± 12.5), while patients with recovery did not (IKDC 65.7 ± 16.7; Lysholm 68.7 ± 16.6). 

In 5 patients with a lesion of the popliteal artery, the result was lower (IKDC 62.6 ± 15.8; Lysholm 70.8 ± 19.9). 

Two patients had a BMI greater than 35 (43.9; 40.1 kg/m^2^) and suffered from ULV. They showed significantly worse outcomes in the IKDC score following ligament reconstruction, compared to patients with BMI lower than 35 (*p* < 0.05; IKDC 36.8 ± 9.8 vs. 67.3 ± 14.28). Lyhsolm Score (*p* = 0.11; Lysholm 54.5 ± 5.0 vs. 71.4 ± 16.3) and TAS (*p* = 0.18 Median 2.5 vs. 4.0) also showed inferior functionality of the knee. They were treated initially in a one-stage procedure. One re-dislocation with consecutive compartment syndrome after ULV, the only one reported in this study, was observed and underwent further surgical treatment because of instability. Arthrofibrosis was documented for the other case and further surgical treatment was required.

### 3.5. External Fixation

Sixteen patients were treated with a knee spanning fixator. In 5 cases it was used because of a traumatic lesion of the popliteal artery, in 2 cases there were severe pelvic fractures, and in 9 cases it was used due to great instability of the knee joint. In 13 out of 16 cases (75.0%) the fixator could be removed after definitive reconstruction. Compartment syndrome occurred in 25.0% of these patients (5% without use of external fixation). Arthrofibrosis occurred twice as often in patients treated with external fixation.

### 3.6. Further Surgical Treatment and Complications

Two-stage treatment, in which the anterior cruciate ligament (n = 15) usually remained untreated, was performed on 16 patients. Only on 6 patients (37.5%) did a ligament reconstruction have to be performed in a further course. The remaining 62.5% patients did not require further surgical treatment due to instability. Short and midterm complications and further surgical treatment are shown in Table 5.

## 4. Discussion

The study results demonstrated a severe limitation of knee function following traumatic knee dislocation in the clinical outcome. The majority of patients did not return to normal knee function (Table 2).

In a systematic review, Marder et al. demonstrated similar clinical outcomes after early reconstruction as those of the current results [40]. 

Timing and technique have been discussed in a recent debate, favoring an early one-staged treatment [30,31]. It was reported that conservative treatment did not lead to satisfying results [41]. Operation within the acute phase of less than 3 weeks after trauma is common, as granulation and scarring, as well as retraction of the ligament stumps. make operations more difficult. Richter et al. demonstrated best clinical results when surgery was performed within the first week [42]. Regarding ligament reconstruction it was reported, that suture repair was equal to ligament reconstruction, while a bracing method could withstand much greater force and was increasingly used for treating the PCL [32]. 

The results of recent studies presenting modern surgical concepts after acute knee dislocation indicated better short-term outcomes [30,31]. However, it is not clear if this is due to ligament augmentation and/or modern rehabilitation programs or other reasons. The poorer mid-term results in this study were partly explained by a more severely injured patient population, as further trauma was not an exclusion criterion. While nerve lesions regenerated in 50% of cases, similar to other studies, lesions of the arteria poplitea required intensive primary operative treatment, such as patches and external fixation. This subgroup is often excluded in knee dislocation studies because the results are inferior (Table 4), and this must be considered when interpreting the results [30,31]. The incidence of injuries to nerves and arteries was similar to other studies [32]. 

Additionally, 20 patients (52.6%) were transferred from other hospitals, due to the complexity of their injuries. It is unclear whether a longer follow-up period would explain the poorer outcomes, as instability and posttraumatic osteoarthritis develop over a longer period. In terms of midterm clinical outcome, patients in this study did not benefit from augmentation of the PCL, in contrast to short-term results [31].

ULV trauma, occurring mostly in obese patients, was considered a different sub=entity, due to the higher complication rate and biomechanical demands of ligament reconstruction, and demonstrated a worse outcome, similar to other reports [31].

The use of external fixation is common in trauma care because immobilization leads to decreased swelling and protects tissues from further damage due to instability. Patients requiring external fixation suffered from severe soft tissue trauma as the rate of compartment syndromes in this group was higher compared to the overall study group (25.0% versus 5.0%). Following external fixation, the incidence of arthrofibrosis was 2 times higher. Therefore, short term application of external fixation might be recommended only if needed, followed by early mobilization and intensive rehabilitation after removal, as this is known to prevent arthrofibrosis [43].

Knee dislocation is known to be a rare and severe injury, usually occurring after traffic or sports accidents, resulting in severely injured patients who present a challenge to therapists in choosing the right time and correct method for reconstruction. In this mid-term follow-up study, knee injury was life-altering, resulting in abnormal knee function and decreased activity levels in the majority of cases.

Further studies, including mid- and long-term results after early ligament augmentation and modern early rehabilitation programs, are needed to demonstrate if knee function can be restored to a higher level. 

### Limitations

The study had limitations, such as its retrospective nature. Another disadvantage was the inherent heterogeneity of the treatment group. The advantages were the exceedingly large cohort size and the fact that all the patients were treated by the same team of surgeons in the same hospital according to the same treatment and aftercare concepts. Considering that knee dislocations are rare, and only a few cases can be found in the literature, the results of this study with a medium-term follow-up of consecutive patients are significant.

## 5. Conclusions

The results of this study indicated frequent abnormal knee function following acute knee dislocation. Similar to other studies, the majority of patients were treated within the first three weeks and with ligament refixation, including PCL augmentation. However, in contrast to other recent studies, the one-stage approach did not show better functional outcomes compared to the two-stage treatment. Additionally, PCL augmentation did not lead to superior results. Inferior outcomes were more likely in obese patients with ULV trauma, and in patients with concomitant traumata or nerve or artery injury. Severe cases with initial external fixation were associated with a higher risk of knee stiffness and this should be considered during rehabilitation. Further prospective multicenter long-term follow-up studies, comparing one-stage versus two-stage therapies are needed to establish a successful treatment concept and to improve clinical outcomes.

## Figures and Tables

**Table 1:** Epidemiological information about the cases studied.

treated patient with knee joint dislocation		120
included		45
excluded		75
lost to follow-up		7 (15.6%)
sex (♀/♂)		11/27
BMI [kg/m^2^]		27.4 ± 4.7
age		49.5 ± 12.7
Follow-up [month]		66.4 ± 32.0
schenck classification	II	1
	IIIM	15
	IIIL	14
	IV	8
		
CPN at trauma		10
persistent CPN at follow-up		5
lesion of a. poplitea at trauma		5
polytrauma/multitrauma/single trauma		3/7/28

**Table 2:** The relevant outcome data from the study group.

	IKDC Score		Lysholm Score	
	M	SD	M	SD
	65.6	±15.7	70.5	±16.4
Grade	*n*	%	*n*	%
A (normal knee function) > 89 points	3	7.9	6	15.8
B (nearly normal knee function) 80–89 points	1	2.6	5	13.1
C (abnormal knee function) 70–79 points	10	26.3	6	15.8
D (severely abnormal knee function) < 70 points	22	57.9	20	52.6
Missing	2	5.3	1	2.6

**Table 3:** Clinical Outcome regarding whether a one-stage procedure, a two-stage procedure or external fixation only was performed. IKDC and Lysholm scores are presented by MD ± SD and the TAS is presented by median. When testing the clinical outcome after external fixation, the results of the one-stage and two-stage approaches were summarized and used for comparison.

	One-Stage Procedure (*n* = 20)	Two-Stage Procedure (*n* = 15)		External Fixation Only (*n* = 3)	
IKDC Score	68.5 ± 17.0	64.0 ± 14.2	*p* = 0.53	50.0 ± 10.6	*p* = 0.10
Lysholm Score	69.9 ± 18.0	73.9 ± 15.7	*p* = 0.42	58.5 ± 60.5	*p* = 0.30
TAS	4	4	*p* = 0.59	4	*p* = 0.72

**Table 4:** Clinical outcome after knee luxation depending on concomitant injuries, such as lesion to nerves or vessels or multiple traumata. Between the 2 groups there was a significant difference (* indicates significant results; *p* < 0.05) regarding the Lysholm score. IKDC and Lysholm scores, presented by MD ± SD, and the TAS is presented by median.

	Mono Trauma (*n* = 20)	Concomitant Injury (*n* = 18)	
IKDC score	69.2 ± 14.6	61.6 ± 16.3	*p* = 0.43
Lysholm score	75.6 ± 15.6	64.3 ± 15.3	*p* = 0.02 *
TAS	4	4	*p* = 0.19

**Table 5:** Further surgical treatment und complications are demonstrated bellow.

	Performed Operation	Number of Cases
Further surgical treatment (*n* = 14; 36.8%)	Arthrolysis	8
	Ligament reconstruction	6
	Conversion Knee Arthroplasty	1
	CPN lesion	2
	Pathology	
Complications (*n* = 18; 47.4%)	Compartment syndrome	5
	Arthrofibrosis	9
	Soft tissue infection	4
	Re-dislocation	1

## Data Availability

There is no supporting data available.

## Abbreviations

IKDC: International Knee Documentation Committee; TAS: Tegner Activity Scale; ACL: anterior cruciate ligament; PCL: posterior cruciate ligament; BMI: Body Mass Index; MV: mean value; SD: standard deviation; HV: high velocity; LV: low velocity; ULV: ultralow velocity; CPN: common peroneal nerve; kg: kilogram; m: meter.
